# Residual Chemical Shift Anisotropies in the Structure Determination of Small Molecules

**DOI:** 10.1002/mrc.70064

**Published:** 2025-12-10

**Authors:** Nilamoni Nath, Juan Carlos Fuentes Monteverde, Swaraj Pathak, Christian Griesinger

**Affiliations:** ^1^ Department of Chemistry Gauhati University Guwahati India; ^2^ Department of NMR Based Structural Biology MPI for Multidisciplinary Sciences Göttingen Germany

## Abstract

The determination of the relative and absolute configuration of natural compounds is a very challenging task. Among other anisotropic NMR parameters, residual chemical shift anisotropy (RCSA) induced by anisotropic media is an invaluable tool to determine relative configurations of natural and synthetic organic molecules in solution. This review introduces various RCSA‐based methodologies for the structural elucidation of natural products. The current availability of alignment media in organic solvents for RCSA measurements is also discussed as are applications of RCSAs for structural analysis of various natural products.

## Graphical abstract

Plasma neutrophil elastase(ELANE) was markedly elevated in patients with alcohol use disorder, indicating a dysregulated protease‐antiprotease inflammatory balance characterised by decreased SERPINA3. ELANE showed excellent diagnostic specificity(AUC = 0.8683), supporting its potential utility as a neuroinflammatory biomarker for AUD. However, ELANE did not predict relapse, emphasising the importance of social and clinical factors in long‐term recovery.

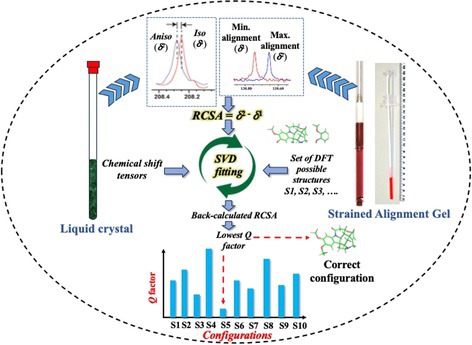

## 1 Introduction

Misassignment of natural products is not uncommon and remains a persistent challenge from the organic chemist's point of view [1, 2, 3, 4]. Although structural misassignments can happen for a variety of reasons, they often originate from high molecular complexity, ambiguity in the resonance assignment, lack of data to investigate the specific structural features, and incorrect interpretation of the available NMR data. Total synthesis of natural products is another key approach for structural assignment, but it can be time‐consuming because multiple stereoisomers must often be prepared [4]. However, assignments derived from total synthesis can sometimes be error‐prone [5, 6, 7]. Single‐crystal X‐ray or electron diffraction structure determination for relative and anomalous diffraction for absolute configuration determination is undeniably a key method in the structure elucidation process. Nevertheless, a well‐diffracting crystal is not always available, and natural products are isolated and purified in amounts that are often too small to screen a whole range of crystallization conditions. Crystalline sponges, on the other hand, provide an attractive potential for some compounds that will not crystallize directly, albeit the sample‐soaking process now requires careful tuning on a per‐molecule basis [8]. In addition, atomic force microscopy has made it possible to visualize the individual atoms, but the molecule must acquire a planar structure for the method to work [9, 10]. Recent advances in electron microscopy can provide a detailed understanding of molecular architecture by revealing the spatial arrangement of atoms in a molecule. Although most of the electron microscopy research has been limited to imaging of biomolecules such as nucleic acids and proteins, advancement in particular in transmission electron microscopy (TEM) including cryo‐EM, in situ TEM techniques, and low electron dose imaging, is enabling direct imaging of small molecules as well [11, 12]. A new method exploiting tensor coupling between the electric and magnetic dipoles has recently been introduced, which holds potential to assign absolute configurations of individual stereocenters [13].

In the arena of spectroscopic methods, NMR is the prime approach for full structural elucidation, especially when crystallization does not work. To begin with structure elucidation, the traditional NMR approaches, notably those reliant on isotropic NMR parameters such as *J* couplings [14, 15, 16, 17, 18, 19, 20] and the nuclear Overhauser effect (NOE) [21, 22, 23, 24, 25, 26], infers the molecular constitution from multiple bits of experimental data. For example, proton and carbon chemical shifts are measured first, providing information on the number of atoms and kinds of functional groups. Following that, scalar (*J*) couplings measured in homonuclear correlation spectroscopy (COSY, E. COSY), heteronuclear single quantum coherence spectroscopy (HSQC), and heteronuclear multiple‐bond correlation (HMBC) establish connectivity between these groups [27, 28, 29, 30, 31, 32]. If mg quantities are available, ^13^C,^13^C‐double‐quantum spectroscopy (ADEQUATE and INADEQUATE) can also be used [16, 33]. Nuclear Overhauser effect spectroscopy (NOESY) and rotating frame Overhauser effect spectroscopy (ROESY) derive distance information between protons, which supports the establishment of spatial connectivities and is required to establish the three‐dimensional (3D) configuration and conformation. To achieve unbiased structure determination, isotropic NMR data together with the molecular formula are loaded into the “computer‐assisted structure elucidation” (CASE) program to unambiguously determine the molecular constitution [34]. Once the molecular constitution has been derived, the next relative configuration and preferred conformation of the molecule are determined. The traditional NOEs, *J* coupling constant analysis, and, in recent times, comparison of ^1^H and ^13^C chemical shifts with the density functional theory (DFT) based values are used for determining the conformation and configuration [32, 35]. However, because of the local character of chemical shifts, *J* couplings, and NOEs, the relative configuration of stereocenters that are distant in the bonding network is oftentimes difficult to determine. It is problematic to solve the structure of flexible molecules, which contain many different conformations due to rotatable bonds. Inversion, for example, at nonplanar nitrogen, is another source of conformational heterogeneity.

The residual dipolar coupling (RDC) [36, 37, 38], residual chemical shift anisotropy (RCSA) [39, 40, 41], and less commonly utilized residual quadrupolar couplings (RQCs) [42] are anisotropic parameters that report on the orientation of internuclear bond vectors, the chemical shift anisotropy tensors, and the interaction of the nuclear quadrupole moment with the local electric field gradient, respectively, with respect to the molecular structure as a whole. They are therefore not local parameters but, in principle, global and able to help determine the relative configuration of stereocenters in rigid molecules, regardless of the distance between them. For flexible molecules, the conformational ensemble needs to be defined to obtain relative configurations. Anisotropic parameters cannot be observed in the normal isotropic solution but need to be induced by deviation from isotropic orientations of the molecules in the solution. This is accomplished by so‐called alignment media, which induce a preferred orientation of the molecules. Such media can be the polymeric gels that are axially or radially compressed or liquid crystals that spontaneously align in the magnetic field due to their intrinsic anisotropic magnetic susceptibility. RDCs are normally derived from heteronuclear internuclear vectors (for organic molecules, preferably CH) and therefore have the experimental sensitivity of proton, carbon 2D correlation spectra. Because of the natural abundance of ^13^C of 1%, this poses challenges to the sensitivity when low amounts of samples are available. RCSAs, on the other hand, specifically proton RCSAs can be detected with the sensitivity of a 1D NMR experiment and are therefore the prime tool to obtain anisotropic parameters in molecules with low amounts (down to approximately 10 μg) available.

Herein, we will present a brief overview of RCSA methodologies for carbon and proton, as well as advancements in alignment media and analysis methods, in the following sections. We will also discuss and summarize the application of RCSA in the structure elucidation of natural products.

## 2 The Chemical Shift and Residual Chemical Shift Anisotropy

In NMR spectroscopy, the observed resonance frequency of a nucleus differs from its Larmor frequency *ω* = *γB*_0_, because of the shielding effects of the surrounding electrons. This electron‐induced shielding effect generates an additional magnetic field (*B*′) that can enhance or reduce the external field *B*_0_, effectively “shielding” the nucleus in case of reduction and “deshielding” in case of enhancement:

(1) $$B_{\mathrm{eff}}=B_{0}-B^{\prime }=B_{0}-\sigma B_{0}.$$

The shielding effect can be described by a so‐called shielding tensor $\hat{\sigma }$, which is a second‐rank tensor and can be mathematically described by a symmetric 3 × 3 matrix. In the laboratory frame, it can be described as

(2) $$\hat{\sigma }=\left(\begin{array}{ccc} \sigma_{\mathrm{xx}} & \sigma_{\mathrm{xy}} & \sigma_{\mathrm{xz}}\\ \sigma_{\mathrm{yx}} & \sigma_{\mathrm{yy}} & \sigma_{\mathrm{yz}}\\ \sigma_{\mathrm{zx}} & \sigma_{\mathrm{zy}} & \sigma_{\mathrm{zz}} \end{array}\right).$$

In this context, each $\sigma_{\mathrm{ij}}$ component of the tensor depends on both the physical and chemical environment of the nucleus, as well as on the molecular orientation. The tensor has three diagonal components, $\sigma_{\mathrm{xx}},\sigma_{\mathrm{yy}},$ and $\sigma_{\mathrm{zz}}$. We take only the symmetric off‐diagonal elements into account: that is, *σ*_*ij*_ = *σ*_*ji*_ because the antisymmetric part of the CSA tensor is nonsecular and only accounts for relaxation effects [43]. The average value of these diagonal elements, $\frac{1}{3}\left(\sigma_{\mathrm{xx}}+\sigma_{\mathrm{yy}}+\sigma_{\mathrm{zz}}\right)$, represents the isotropic chemical shielding, which remains constant regardless of the reference frame, providing an orientation‐independent measure of the average shielding effect of the nucleus.

Conventionally, *B*_0_ is represented by a vector ( $\vec{B_{0}}$), producing the shielding‐dependent $\vec{B^{\prime }}$. For simplicity and because it is a real symmetric tensor, without reduction of generality, we diagonalize the shielding tensor, which means that we represent it in its eigenframe, yielding

(3) $$\vec{B^{\prime }}=\left(\begin{array}{ccc} \sigma_{\mathrm{xx}} & 0 & 0\\ 0 & \sigma_{\mathrm{yy}} & 0\\ 0 & 0 & \sigma_{\mathrm{zz}} \end{array}\right)\vec{B_{0}}=\left(\begin{array}{ccc} \sigma_{\mathrm{xx}} & 0 & 0\\ 0 & \sigma_{\mathrm{yy}} & 0\\ 0 & 0 & \sigma_{\mathrm{zz}} \end{array}\right)\left(\begin{array}{c} b_{0x}\\ b_{0y}\\ b_{0z} \end{array}\right)=\left(\begin{array}{c} \sigma_{\mathrm{xx}}b_{0x}\\ \sigma_{\mathrm{yy}}b_{0y}\\ \sigma_{\mathrm{zz}}b_{0z} \end{array}\right).$$

Because $\sigma_{\mathrm{xx}}\;\text{and}\;\sigma_{\mathrm{yy}}$ differ from $\sigma_{\mathrm{zz}}$, $\vec{B^{\prime }}$ is not parallel to $\vec{B_{0}}$, yet only the parallel component of $\vec{B^{\prime }}$ to $\vec{B_{0}}$ contributes due to the factor 10^4^–10^5^ size difference of $\vec{B^{\prime }}$ and $\vec{B_{0}}$. Therefore, the orientation‐dependent chemical shift is given as follows, with $\vec{B_{0}}$ represented by the Cartesian components: $\left(b_{0x},b_{0y},b_{0z}\right)$.

(4) $$\sigma_{\text{aniso}}=\vec{b_{0}^{T}}\sigma \vec{b_{0}}=\;b_{0x}^{2}\sigma_{\mathrm{xx}}+b_{0y}^{2}\sigma_{\mathrm{yy}}+b_{0z}^{2}\sigma_{\mathrm{zz}}$$

Clearly, as the molecules tumble in solution, the orientation of the external magnetic field relative to the tensor frame (TF) changes stochastically. In isotropic media, the probability of the different orientations of the *B*_0_ field is identical, namely, $\frac{1}{3}$. In an anisotropic medium, however, different probabilities for the three orientations $\left(p_{x},p_{y},p_{z}\right)$ are observed, resulting in the anisotropic contribution to be

(5) $$\sigma_{\text{aniso}}=\left(\left(p_{x}-\frac{1}{3}\right)\sigma_{\mathrm{xx}}+\left(p_{y}-\frac{1}{3}\right)\sigma_{\mathrm{yy}}+\left(p_{z}-\frac{1}{3}\right)\sigma_{\mathrm{zz}}\right).$$

These excess probabilities $\left(p_{i}-\frac{1}{3}\right)$ are the diagonal components of the alignment tensor $A_{\mathrm{ij}}$. This leads to the equation for the chemical shift in arbitrary reference frames [44]:

(6) $$\left\langle \sigma \right\rangle =\sigma_{\text{aniso}}+\sigma_{\mathrm{iso}}=\sum_{i,j}\sigma_{\mathrm{ij}}A_{\mathrm{ij}}+\frac{1}{3}\left(\sigma_{\mathrm{xx}}+\sigma_{\mathrm{yy}}+\sigma_{\mathrm{zz}}\right).$$

The first term represents the anisotropic shielding ( $\sigma_{\text{aniso}}$) and the second term is the isotropic shielding ( $\sigma_{\mathrm{iso}}$). The term $\sigma_{\text{aniso}}$ arises because of the RCSA effect. It should be mentioned that in liquid‐state NMR, one rather uses deshielding (*δ*) instead of shielding (*σ*). The two quantities are the same except for the sign.

It was until 2011 that RCSAs could not be used as a complementary method to RDCs for determining the relative configuration of many natural products. The RCSA measurement has the advantage over RDCs as the chemical shift is the simplest parameter to measure in NMR, and it can be measured from 1D ^13^C‐{^1^H} NMR or ^1^H NMR spectra. The RCSA could only be induced by an alignment medium, and in principle, it can be obtained as a chemical shift difference between the aligned and nonaligned samples. Such measurements suffer from the effect of isotropic shift as the sample environment's changes when going from isotropic to anisotropic phases. It is also difficult to separate the isotropic shift effects from the desired RCSA effects. Therefore, samples amenable to RCSA measurements need to be far better controlled so that the sample's condition changes very minimally from no alignment to alignment. Therefore, one strategy to accurately measure RCSAs is to conduct the experiments using the same aligned sample under two different alignment conditions as in the case of stretched polymer gel and in some liquid crystal measurements [29, 40, 41, 45, 46]. In an alternative method, the changes in isotropic shifts are calculated for compressed gel and in some liquid crystal–based RCSA measurements [41, 47, 48].

### 2.1 Anisotropic/Alignment Media

The first ^1^H NMR spectrum of a partially aligned organic molecule, benzene in a nematic liquid crystalline phase, was reported by Saupe and Englert [49]. The large alignment strength of the liquid crystal leads to distinct chemical shifts for each benzene's proton, and dipolar couplings are larger than the chemical shift differences, resulting in strongly coupled spectra [50]. Therefore, weak alignment media are introduced for RDCs, which result in first‐order spectra of the analyte molecule, and they can also be used for the collection of RCSA data. Presently, two types of weak alignment media are available for measurements of RCSAs: lyotropic liquid crystals (LLCs) [51, 52, 53, 54, 55] and chemically cross‐linked gels (CCGs) [41, 48, 56, 57]. The LLC phases have a larger anisotropy because of the magnetic susceptibility and therefore, they align spontaneously in the same direction in the presence of a strong external magnetic field, and this alignment is partially transmitted to the solvent and molecules in solution. In many polymeric LLC phases, the alignment strength can often be tuned over a wide range, simply by adjusting the temperature. By contrast, for cross‐linked gels, the anisotropy is formed mechanically through the compression or stretching of the gels, which provides a direct means of modulating the degree of alignment.

### 2.2 Liquid Crystals

The use of LLCs different from PBLG and similar alignment media was proposed in recent years for RCSA measurement [52, 58]. The LLC phase of PBLG is a good choice for RCSA measurement because of its ability to induce stronger alignment. This medium was extensively used for enantiodiscrimination studies using proton, carbon, and deuterium NMR [59, 60, 61, 62, 63, 64, 65, 66, 67, 68, 69]. The PBLG liquid crystal was utilized for RCSA measurement based on the linear dependence of chemical shifts with PBLG concentration [51]. The liquid crystal based on AAKLVFF [53] oligopeptides, biphasic C_21_H_43_–CONH‐V_4_K_3_–CONH_2_ [55], and biphasic L‐valine‐derived helically chiral polyacetylenes (PLAs) [52], has been developed for RCSA measurement. Another liquid crystal based on (FK)_4_ oligopeptides [54], giving rise to tunable anisotropic and isotropic phases through temperature, has been used to measure both RDCs and RCSAs. The AAKLVFF peptide, which forms liquid crystals, was the first instance of employing a MeOH‐based LLC as an alignment medium at a low concentration. Further, four known natural compounds with widely varying functional groups were assessed to show the wide compatibility and usefulness of RCSA analysis. Remarkably, the structure of spiroepicoccin A [53, 70], an unknown novel marine natural product isolated from the deep‐sea derived fungus *Epicoccum nigrum*, was elucidated. Later, this LLC was successfully utilized to elucidate the correct structure of the novel, weizhouochrone A [71]. The configuration could be assigned by a combination of both RDC and ^13^C‐RCSA.

In addition to low critical concentrations, several factors determine the suitability of LCCs for RCSA measurements. These include background signals in the NMR spectrum, line broadening, sample homogeneity, and compatibility with the analyte. An essential feature is the coexistence of isotropic and anisotropic phases in the NMR tube, which enables the collection of RCSA data in a single experiment. The extent of alignment is another decisive factor, whereas the kinetics of phase formation—ranging from minutes in PLAs to several weeks in self‐assembling peptides—also plays a critical role.

The unmodified graphene oxide (GO)–based LLCs are recently discovered alignment media that act as unique anisotropic orientation media with a low critical concentration and easy scaling. They met the criteria for an ideal orientation medium. Because of the stiffness and high molecular weight of GO molecules, they may induce anisotropic interactions to align tiny molecules, which has unanticipated advantages, such as the unprecedented feature of high‐quality NMR spectra without any background signals. Recently, GO‐derivatized cyclopentylamine (GO‐d‐CP) liquid crystals, which overcome the demerits of other GO‐based liquid crystals, have been used to measure RCSAs, and measured RCSAs were utilized to solve the configurations of several molecules [45]. The GO‐d‐CP liquid crystal is also used to determine correct regioisomers of a spiro compound and the correct isomer of a breitfussin analogue [72]. It is easy to synthesize, easy to obtain as a solid, and easy to achieve the liquid crystalline state. It offers a stable anisotropic state for prolonged periods and allows analyte recovery and recovery of the GO‐d‐CP itself for reuse.

### 2.3 Strain‐Induced Gels (SAGs)

SAGs are a reliable method for measuring RCSAs. SAGs are either CCGs or gels cross‐linked by irradiation with accelerated electrons, creating covalent bonds during the polymerization reaction. The gels need to swell in solvent to create anisotropic cavities in which the solute aligns [73]. The cross‐links of the gels create a stable monodomain net to keep the structural integrity of the gel even when it swells in the presence of organic solvents. Under radial or axial compression, the cavities formed within the SAG adopt an ellipsoidal shape, where they can be described as either oblate (flattened along the *z*‐axis under radial compression) or prolate (elongated along the *z*‐axis under axial compression), with the main symmetry axis aligned with the external magnetic field *B*_0_. SAGs have a director consistently throughout the sample [74], which effectively and uniformly causes alignment of the molecules. In this sense, they behave as a homogeneous medium, like conventional LLCs [75]. For accurate determination of the anisotropic NMR parameters, maintaining sample homogeneity is essential. Conventionally, this is checked using a simple 1D ^2^H NMR spectrum, where symmetric quadrupolar splitting indicates that the sample is ready for measurement. However, the ^2^H spectrum is not always clean or symmetric because of the variation in alignment across the sample volume. In such a scenario, ^2^H imaging of partially aligned samples provides a means of visualizing sample homogeneity with respect to both the magnetic field and alignment strength [76, 77]. We first describe the chemical composition of the gels and then the devices that deform them either radially or axially. The first documented/reported gel, the polystyrene (PS) gel, cross‐linked with divinylbenzene, was used as an alignment medium for small organic molecules, which was compatible with organic solvents [78]. It was first swollen in CDCl_3_ and stretched inside a 5.0‐mm NMR tube. In the 5‐mm NMR tube, a PS polymer rod with a diameter of approximately 4 mm and a length of 10 mm was inserted. When CDCl_3_ was added to the NMR tube, the gel expanded until it met the tube's diameter, yet the gel continued to swell vertically, extending itself. The swelled gel occupied a volume of roughly 4 cm in height in the NMR tube. During the swelling phase, a sample solution in the deuterated solvent can be gently diffused into the gel. The degree of alignment cannot be adjusted after the gel has expanded. However, with this procedure, the degree of alignment may be adjusted by changing the gel's cross‐link density or the diameter of the polymer rod. This work was the beginning of what is now a highly effective approach for aligning and measuring anisotropic parameters. Soon after, different types of polymeric gels compatible with a range of solvents were discovered. Even special devices were developed to either stretch or compress gels. Recently, PS gel was used for accurate measurements of ^1^ *D*_CH_ and ^13^C‐RCSAs in various solvents such as toluene‐*d*_8_, pyridine‐*d*_5_, and CDCl_3_ [79].

One of the approaches for stretching gels involved using a fluorinated elastomer (Kalrez 8002) [80], which has high resistance to most chemicals, offers high purity, and is compatible with organic solvents. Later, a stretching device, which was originally designed for measuring RCSAs of biomolecules [81], was adopted for measuring RCSAs in small molecules using organic solvent compatible with various gels such as cross‐linked, poly (methyl methacrylate) (PMMA) [41], poly‐(2‐hydroxylethyl methacrylate) (poly‐HEMA) [56] gel, and a fully deuterated version of PMMA‐*d*_8_ [58], the latter being compatible with the microstretching device (MSD). The other approach is based on the reversible compression/relaxation of the gel. The CCGs used for measuring RCSAs using this method include PMMA gels swollen in CDCl_3_ [41], polyacrylonitrile (PAN) gel swollen in DMSO‐*d*_6_ [57], and PMMA‐*d*_8_ swollen in CD_2_Cl_2_ [32]. All these CCGs are compatible with 5‐ and 3‐mm compression devices. Several polymer gels have also been reported for observing RDC; however, they should also be applicable to RCSA protocols, namely, stretched PAN gels [82], universal poly (ethylene oxide) gels [83], poly‐2‐acrylamido‐1‐propanesulfonic acid (APS) gel, and polyurethane (PU) gels [84]. Most of the CCGs are washed immediately after preparation by radical chemical cross‐linking reaction to remove the residual monomer and oligomers. Cross‐linking with accelerated electrons is reported to produce monomer‐free gels [85].

Generally, the sample can be washed out from the gels using the same solvent used for swelling the gel, and the gel stick can be used again several times. Sample recovery is expected to be around 80% of the initial mass. The approach using polymeric gels is one of the most straightforward methods in terms of sample preparation, homogeneity, and reproducibility [76].

The proportion of monomer and cross‐linker is a major point to consider during gel formation, as it has a direct impact on their mechanical properties and swelling capabilities. A strained gel generates anisotropy in the analyte by physical deformation, either by compression or stretching. Compression‐compatible gels are commonly prepared with monomer concentrations around 80% v/v and a cross‐linker concentration between 0.1 and 0.7% molar. The actual monomer/cross‐linker ratio might vary depending on their characteristics. For example, in the preparation of poly‐HEMA, where the cross‐linker is ethylene glycol dimethacrylate (EGDMA), it should be kept as 80/0.27, whereas for the preparation of PAN, the ratio used is 67/0.7, and the cross‐linker is ethylene glycol diacrylate (EGDA). As a rule of thumb, it seems that a radical initiator concentration of 10 mol% of the cross‐linker is a good starting point whenever a new formulation of CCGs is being developed [86]. This concentration makes a gel rigid enough to handle compression without collapse, whereas the level of crosslinking allows fine‐tuning of anisotropy when compression is applied [57]. In contrast, if the CCG is meant to be stretched, the resulting stick needs to be more elastic while maintaining its flexibility. This is achieved by using monomer concentrations in the range of 70% v/v and CL concentrations in the range of 0.1%–2.0% molar [87, 88]. This formulation allows the gel to adopt the needed shape under the action of a longitudinal force without collapsing and inducing a homogeneous alignment of the sample molecules inside the polymer net. Selection of monomer and cross‐linker concentration, together with the kind of device employed (compression or stretching), is crucial to obtain precise and reproducible RCSA data. The main challenge is to induce small or zero isotropic chemical shifts in both isotropic and anisotropic conditions, whether in compression or stretching devices. We have provided an overview of alignment media and will now describe how RCSAs are measured.

## 3 RCSA Measurement Strategies

### 3.1 Variable Angle Spinning

Isotropic chemical shifts are not affected by magic angle sample spinning (MAS). The RCSA, on the other hand, is scaled by $3\cos^{2}\theta -1$, when spinning about an axis with the angle *θ* to the *B*_0_ field direction is chosen. The first ^13^C RCSA measurement was achieved for small molecules in a weak alignment medium using a variable angle probe (VASS) [39, 89]. They have utilized a conventional MAS rotor to prepare the anisotropic sample, where anisotropic conditions are induced by changing the angle of the director of the alignment to the static magnetic field. Rotor heads of 5 and 4 mm with a glass tube of 4 mm were used. As an alignment medium for the proof of concept, PDMS gel swollen in CDCl_3_ was used. A. Saupe demonstrated, in 1964, that the size and sign of anisotropic interactions in a liquid crystalline phase may be scaled by adjusting the angle *θ* of the director of the mesophase relative to the static magnetic field *B*_0_ [90]. Accordingly, they altered the amplitude of the measured anisotropic parameters using the $\frac{3}{2}\cos^{2}\theta -\frac{1}{2}$ dependence. The same relationship holds true for stretched gel alignment, which requires varying the angle of the stretching axis relative to *B*_0_. The VASS probe is built such that radiofrequency irradiation always has a component perpendicular to the *B*_0_ field at all angles *θ* of the sample axis to the external field. They observed the deuterium splitting for CDCl_3_. They were able to confirm the $\frac{3}{2}\cos^{2}\theta -\frac{1}{2}$ dependence over the whole range of *θ* from 0° to 90°, especially confirming the vanishing of chemical shift anisotropy at the magic angle (54.7°), yet the resolution was poor. The probe was switched to a 4‐mm MAS probe, where they could achieve reasonable ^13^C linewidths.

### 3.2 Gel Stretching and Gel Compression

As discussed in the beginning, the observed chemical shift $\left(\delta \right)$ in an anisotropic environment consists of two components, an isotropic term of an arbitrary nucleus *i*, $\delta_{i}^{\mathrm{iso}}$, and an anisotropic term, ${\text{RCSA}}_{i}$. To extract the RCSA from the isotropic chemical shift, we need two different alignment conditions. Because $\delta_{i}^{\mathrm{iso}}$ is highly sensitive toward the chemical environment, and even small environmental differences between the alignment conditions lead to inaccurate RCSAs. Therefore, parameters like pH, solvent, analyte concentration, and sample temperature must be kept constant. Compensation for analyte concentration and sample temperature has been introduced in RCSA protocols [41, 48, 52]. For accurate RCSA measurement, a setup is required in which $\delta_{i}^{\mathrm{iso}}$ remains either constant or predictably varies for the two alignment conditions, enabling its removal during post‐processing. For example, the variable angle NMR technique [39] has been proven to be an effective method for RCSA measurement by leveraging the ability to modulate anisotropic NMR data simply by adjusting the alignment angle relative to the magnetic field without affecting the chemical environment, that is, $\delta_{i}^{\mathrm{iso}}$. However, this method requires specialized tools that are not widely available in most laboratories and has the limitations described in the previous paragraph.

**FIGURE 1:**
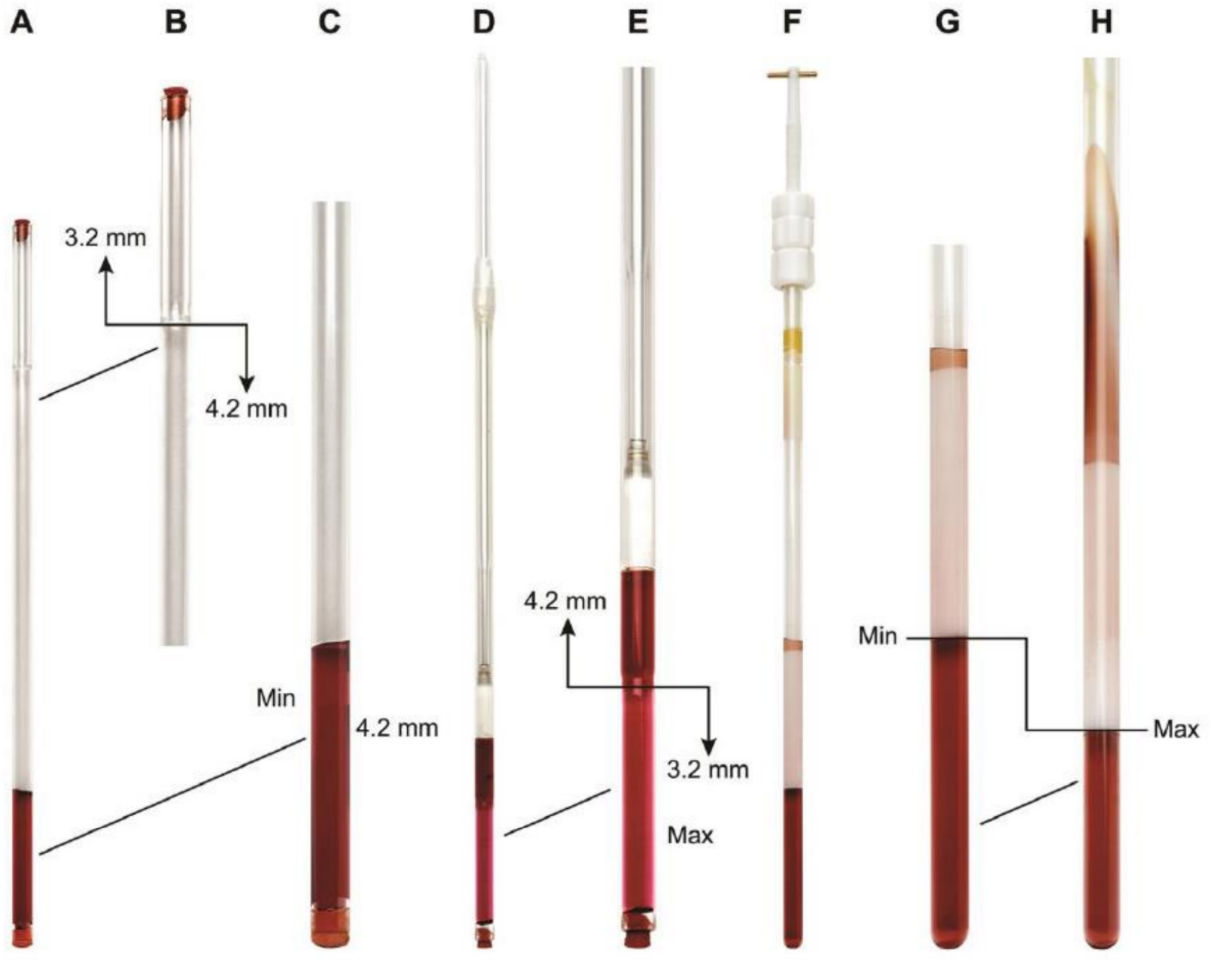
Photographs of the stretching and compression devices. The PMMA gel used in this figure is colored for visualization with the violet‐colored alkaloid Cryptolepine dissolved in deuterochloroform. Panels A–E pertain to the tube designed for stretching the gel. Panels F–H show the apparatus for compressing the gel used for the RCSA measurements. (A) Tube with the PMMA gel positioned in the 4.2‐mm‐diameter section of the NMR tube. Rubber stoppers are used to close the top and bottom of the tube. (B) Expansion showing a close‐up of the top of the tube from Panel A. The line indicates the location where the inner diameter of the tube changes from 3.2 to 4.2 mm. The 3.2‐mm‐diameter section of the tube is used for maximum stretching, whereas the 4.2‐mm‐diameter section of the tube gives minimum stretching of the gel. (C) Close‐up of the tube with the gel in the 4.2‐mm‐diameter section of the tube. (D) Full tube with the gel stretched in the 3.2‐mm‐diameter segment of the tube. The difference in color defines where the inner diameter changes from 3.2 to 4.2 mm. The narrower segment is lighter in color than the larger ID segment and is centered on the radiofrequency coil of the probe. (E) Close‐up of the segment of the tube with the gel in the 3.2‐mm‐diameter section. The difference in color where the inner diameter of the tube changes is more readily visible in this panel. (F) Photograph of the full assembly used for compressed gel sample measurements. (G) Close‐up showing the gel in the minimum compression condition. (H) Close‐up showing the gel in the maximum compression condition. Panels G and H are scaled identically. The difference in the vertical dimension corresponds to the difference in compression of the gel in the minimum and maximum compression conditions [41]. (Permission expected (ref. 41).)

Soon afterward, Hallwass et al. utilized the rubber stretching device (Kuchel apparatus) [91] for RCSA measurement using a DMSO‐compatible (S)‐2‐acrylamido‐propanesulfonic acid (APS) gel [40].

(7) $$\begin{array}{c} \Delta {\text{RCSA}}_{k}\left(\mathrm{ppm}\right)=\left[\left\{\delta^{\left(1,k\right)}-\delta^{\left(1,\mathrm{ref}\right)}\right\}-\left\{\delta^{\left(2,k\right)}-\delta^{\left(2,\mathrm{ref}\right)}\right\}\right]\\ =\sum_{i=x,y,z}\sum_{j=x,y,z\;}\left[\left(A_{\left(\mathrm{ji}\right)}^{\left(1\right)}-A_{\left(\mathrm{ji}\right)}^{\left(2\right)}\right)\left(\;\delta_{\left(\mathrm{ji}\right)}^{{\mathrm{CSA}}_{k}}-A_{\left(\mathrm{ji}\right)}^{{\mathrm{CSA}}_{\mathrm{ref}}}\right)\right] \end{array},$$

where $A_{\left(\mathrm{ji}\right)}^{\left(1\right)}$ and $A_{\left(\mathrm{ji}\right)}^{\left(2\right)}$ are the alignment tensors that represent the two degrees of stretching of the gel inside the stretching device. It was ensured that the conditions affecting the isotropic shifts were the same. During stretching, the overall magnetic susceptibility also gets modified, which, in turn, causes changes in the resonance frequency of all nuclei. This is removed either by taking a nucleus of the analyte as an internal reference or a tetrahedral molecule such as CCl_4_ or TMS [92, 93]. Because there is no isotropic shift effect, ΔRCSA is obtained by subtracting the term $\left\{\delta^{\left(2,k\right)}-\delta^{\left(2,\mathrm{ref}\right)}\right\}$ from $\left\{\delta^{\left(1,k\right)}-\delta^{\left(1,\mathrm{ref}\right)}\right\}$ between maximum and minimum alignment conditions taking any carbon as a reference. Later in 2016, Nath et al. used the stretching device as shown in Figure 1A–E.

Although gel compression has been in use for RDC measurement for quite some time, RCSA data collection from the compressed gel was realized only in 2016. This is mainly due to the difficulty in isolating the contribution of isotropic shift effects on RCSAs. Nath et al. tried to achieve two alignment conditions using these compression devices [41]. The same active volume is employed in the compression technique for both strong and weak alignment, resulting in equal signal‐to‐noise for both scenarios. However, the analyte concentration inside the gel stick changes during compression. In this device, a portion of the analyte with the solvent always stays outside the gel. The internal surface area is constant, and on compression, the inner liquid volume decreases with compression, causing a rise in the gel‐to‐analyte ratio and an isotropic shift change for the analyte positioned in the gel, contaminating the RCSA measurement. Therefore, for accurate RCSA data, they eliminated the isotropic shift effect. They formulated RCSA as

(8) $${\text{ΔRCSA}}_{i}\left(\mathrm{ppm}\right)=\left[\left(\delta_{i}^{\max}-\delta_{\mathrm{ref}}^{\max}\right)-\left(\delta_{i}^{\min}-\delta_{\mathrm{ref}}^{\min}\right)\right]-\frac{{\bar{\left(\delta_{i}^{\max}-\delta_{\mathrm{ref}}^{\max}\right)-\left(\delta_{i}^{\min}-\delta_{\mathrm{ref}}^{\min}\right)}}^{\mathrm{sp}3}}{{\bar{\left(\delta_{i}^{\min}-\delta_{\mathrm{ref}}^{\min}\right)-\left(\delta_{i}^{\mathrm{iso}}-\delta_{\mathrm{ref}}^{\mathrm{iso}}\right)}}^{\mathrm{sp}3}}\left[\left(\delta_{i}^{\min}-\delta_{\mathrm{ref}}^{\min}\right)-\left(\delta_{i}^{\mathrm{iso}}-\delta_{\mathrm{ref}}^{\mathrm{iso}}\right)\right],$$

where $\frac{{\bar{\left(\delta_{i}^{\max}-\delta_{\mathrm{ref}}^{\max}\right)-\left(\delta_{i}^{\min}-\delta_{\mathrm{ref}}^{\min}\right)}}^{\mathrm{sp}3}}{{\bar{\left(\delta_{i}^{\min}-\delta_{\mathrm{ref}}^{\min}\right)-\left(\delta_{i}^{\mathrm{iso}}-\delta_{\mathrm{ref}}^{\mathrm{iso}}\right)}}^{\mathrm{sp}3}}$ is the isotropic shift correction factor “*c*.”

Both approaches, stretching and compression, were evaluated with small molecules aligned in chloroform‐compatible PMMA gels for this study, but the same tools may also be used to restrict gels compatible with other solvents.

Similar approaches for compressing data had been made by Hallwass and his coworkers [48]. They used a similar equation, which was originally proposed by Nath et al. to extract RCSAs from compressed gel. However, they pointed out that the isotropic shift correction factor, “*c*,” might be difficult to obtain for molecules where there are very few sp^3^ carbons. Therefore, to mitigate the problem and make it user‐friendly, the linear scaling parameter is automatically optimized along with the alignment tensor of the fitting procedure itself while still retaining configuration discrimination capability.

### 3.3 Concentration‐Dependent RCSA Extraction

RCSAs are measured for biomolecules using different percentages of liquid crystals by estimating solvent effects [94]. By adopting a similar protocol, Liu et al. introduced a simple and generally successful technique for accurately quantifying RCSAs in the PBLG mesophase for the determination of the structure of small molecules [47]. Data were acquired for caulamidine A, retrorsine, and strychnine, and compared with the RCSA data from stretched PMMA or poly‐HEMA gels available in the literature. For reliable RCSA measurement, the challenge is to remove Δ*δ*_iso_ related to the PBLG phase transition at different concentrations from isotropic to anisotropic. The technique assumes that the Δ*δ*_iso_ dependency on PBLG concentration is the same in both phases; therefore, the Δ*δ*_iso_ in the mesophase may be compensated by extrapolating its value from the concentration dependence in the isotropic phase. Experimentally, they introduced modest quantities of PBLG to a strychnine solution one at a time, acquiring a ^13^C‐{^1^H} spectrum after each addition. They added small amounts of PBLG to the strychnine solution and recorded the ^13^C‐{^1^H} spectrum after each addition. TMS reference eliminates the influence of the bulk susceptibility shift at different PBLG concentrations. Otherwise, referencing for RCSA measurements can be done using an arbitrary carbon of the analyte. PBLG was gradually added to the solution, and the deuterium quadrupolar splitting (^2^H‐RQC) was measured after each addition consistent with an isotropic solution. When the PBLG critical concentration is reached (*C*_crit_: 11.4%), a substantial downfield resonance leap occurs owing to RCSA in conjunction with a ^2^H‐RQC of 215.5 Hz in the CDCl_3_ signal, indicating that the sample had entered the mesophase. This behavior can be used for a linear correction using Equation (9):

(9) $${\text{ΔRCSA}}_{i}\left(\mathrm{ppm}\right)=\left(\delta_{\text{aniso}}^{i}-\delta_{\text{aniso}}^{\mathrm{TMS}}\right)-\left(\delta_{\mathrm{iso}-0}^{i}-\delta_{\mathrm{iso}-0}^{\mathrm{TMS}}\right)-\frac{{\left[\text{PBLG}\right]}_{\text{aniso}}}{{\left[\text{PBLG}\right]}_{\mathrm{iso}-1}}\left[\left(\delta_{\mathrm{iso}-1}^{i}-\delta_{\mathrm{iso}-1}^{\mathrm{TMS}}\right)-\left(\delta_{\mathrm{iso}-0}^{i}-\delta_{\mathrm{iso}-0}^{\mathrm{TMS}}\right)\right].$$

$\delta_{\mathrm{iso}-0}^{i}$ is measured in an isotropic phase without PBLG, whereas $\delta_{\mathrm{iso}-1}^{i}$ is obtained from a second isotropic measurement with a PBLG concentration slightly less than *C*_crit_ and $\delta_{\text{aniso}}^{i}$ is an anisotropic measurement at a PBLG concentration above *C*_crit_. It is important to note that all the terms on the right side of Equation (9) could be experimentally determined.

### 3.4 Magnetically Induced RCSA

Magnetically induced alignment or self‐alignment is a phenomenon that achieves alignment without the use of an alignment medium. One effective method for generating anisotropic conditions is to leverage the alignment induced by an external magnetic field. This self‐alignment is produced for the analyte when it exhibits anisotropic magnetic susceptibility. The extent of this alignment scales with the square of the external magnetic field, *B*_0_. In the case of small molecules, aromatic systems are the most significant contributors to anisotropic magnetic susceptibilities. Usually, field‐dependent experiments are performed to measure the RQC, RDCs, and RCSAs; however, the effects observed are generally considerably weaker compared to those seen in paramagnetic metal centres, where a significant degree of alignment is usually observed because of the high anisotropic susceptibilities [95].

Karschin et al. were the first to report the use of the self‐alignment method for measuring RDC and RCSA for small‐molecule structure determination [96]. The feasibility of the methodology was checked using two molecular systems: The first was strychnine, a well‐known alkaloid with a single aromatic ring. The second was gymnochrome G [97, 98], an aromatic system consisting of eight fused rings, resulting in a high degree of alignment. Despite its low degree of self‐alignment, the method enabled them to unambiguously determine the correct configuration from its set of diastereomers, highlighting the versatility and effectiveness of the technique. Because the anisotropic component of the chemical shift value directly depends on the square of the magnetic field value, a straightforward fitting procedure was employed to determine the required anisotropic component. The methodology involves the correction of measured chemical shifts by removing the systematic errors occurring due to temperature fluctuations, degradation effects, and referencing inconsistencies. For RCSAs, the corrected chemical shifts were then converted to peak positions in Hertz, and the measured frequency was expressed as the following equation:

(10) $$v_{\text{meas}}=\delta_{\mathrm{iso}}v_{\mathrm{ref}}+\delta_{\text{aniso}}v_{\mathrm{ref}}B_{0}^{2}=\delta_{\mathrm{iso}}\gamma B_{0}+\delta_{\text{aniso}}\gamma B_{0}^{3}.$$

Here, both the isotropic and anisotropic terms appear in the linear form with respect to the magnetic field, allowing the application of least squares fitting to resolve these parameters from a given set of experimental data ( $v_{\text{meas}}$, $B_{0}$). They also used the same protocol for the measurement of the anisotropic component in RDC evaluation.

### 3.5 Biphasic Liquid Crystals: PBLG and Polyacetylene

To determine RCSAs using aligned media, there are three ^13^C NMR spectra mainly required: one from an isotropic solution, another from an isotropic state in the alignment medium, and a third from an aligned state. By using these experimental conditions, RCSA values were obtained by observing the differences in chemical shifts under two alignment conditions: minimum and maximum alignment, respectively. This approach allowed for the isolation of the isotropic chemical shifts, although potential experimental errors could arise because of instrument variations and specific experimental conditions. Thus, to avoid the need for repeated spectral acquisitions under two conditions, new biphasic liquid crystal media using PBLG [99], L‐valine‐derived polyphenylacetylene [52], and oligopeptide developed using self‐assembly of C_21_H_43_–CONH‐V_4_K_3_–CONH_2_ [55] have been developed. This “one‐shot” RCSA measurement approach offers faster analysis, reduced material use, self‐referencing, and high accuracy. There are other bimesophasic liquid crystals, for which RQCs were measured, that can be in principle used for RCSA measurements [100, 101].

**FIGURE 2:**
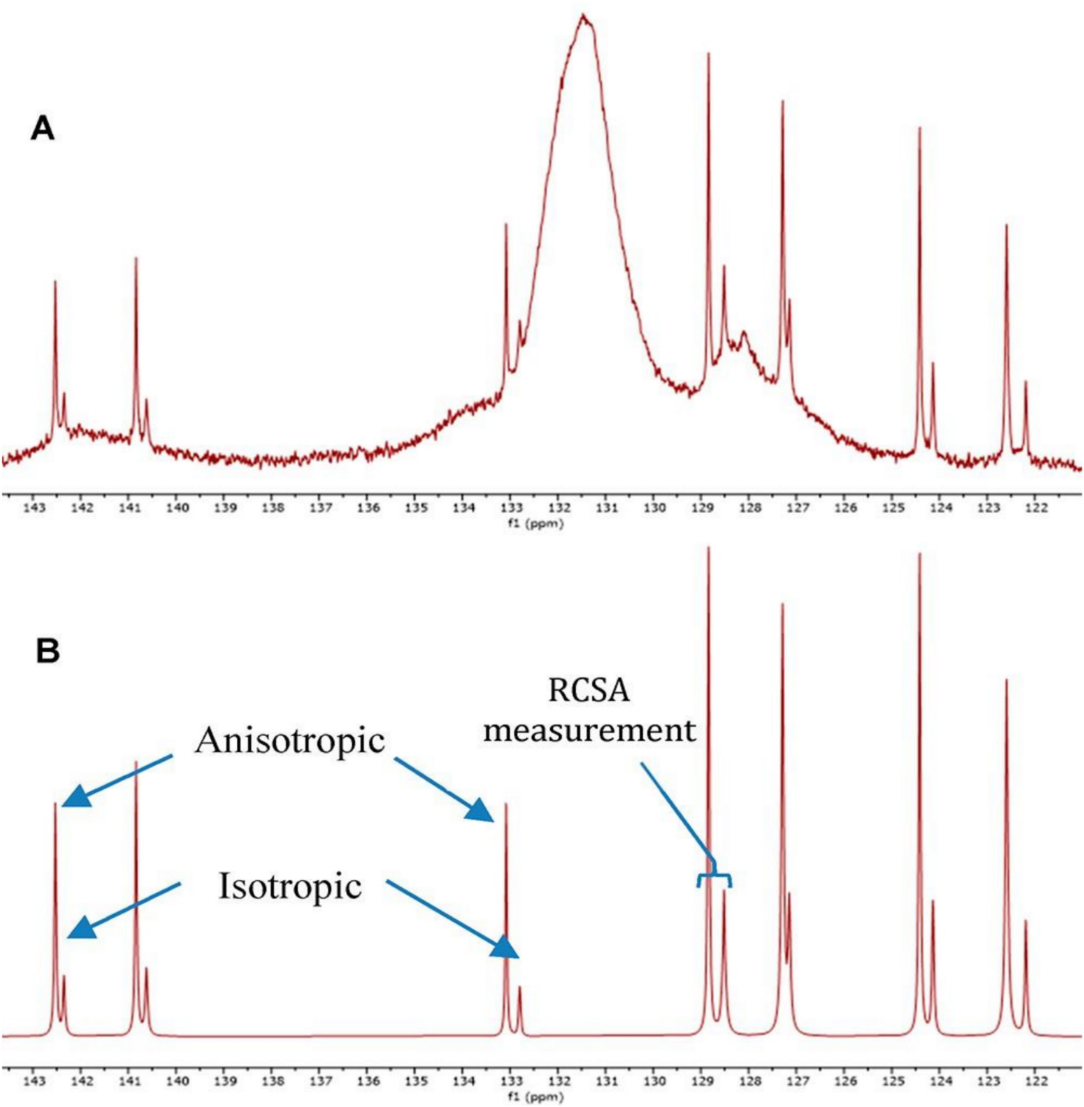
Aromatic region expansion of the one‐dimensional (1D) ^13^C‐{^1^H} NMR spectrum (150.9 MHz) of strychnine in PBLG showing NMR resonances for seven sp^2^ carbons and their respective isotropic and anisotropic NMR resonances. Native 1D ^13^C NMR data. (A) 1D ^13^C NMR data processed with global spectral deconvolution (B) [99]. (taken from ref. 55. Permission expected.)

In the case of biphasic PBLG in CDCl_3_, Racchia et. al induced the biphasic behavior through spectroscopic titration, varying concentrations of PBLG across two molecular weights of the LLC [99]. Initially, the LLC exhibited an isotropic dispersion, but just before reaching a fully anisotropic phase, a mixed isotropic–anisotropic phase was observed. They also noticed that the lower‐molecular‐weight PBLG required higher concentrations to form the LLC phase but yielded stronger alignment once anisotropy was established. Further, they utilized this biphasic phase to evaluate the RCSA data to evaluate the configuration of strychnine and a conformational study on neotricone. The following figure showcasing two distinct resonance subsets for strychnine appears in the biphasic phase (intermediate) of the PBLG‐LLC, where one is from an isotropic solution and the other from an anisotropic solution (Figure 2A). The simultaneous extraction of ^13^C chemical shift in both isotropic and anisotropic environments is facilitated when data is processed with global spectral deconvolution (GSD) (Figure 2B). This approach allows ^13^C RCSA extraction within a single experiment, eliminating the need for an external reference and the traditional three‐step measurement process. Given its simplicity, this methodology is attractive when it is required to study small molecules using LLC.

**FIGURE 3:**
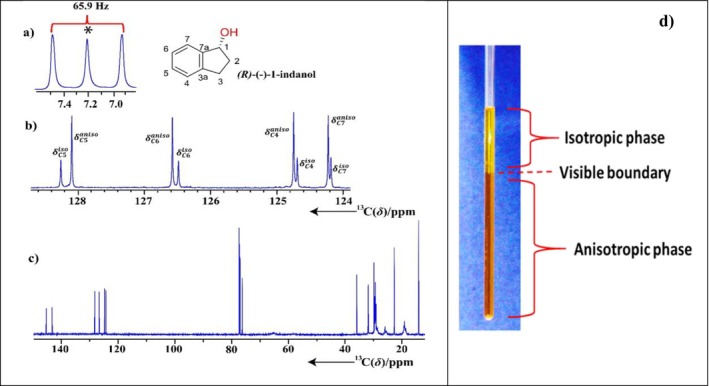
1D ^2^H NMR spectrum of CDCl_3_ in the (R)‐(‐)‐1‐indanol sample aligned in the LLC phase of L‐valine‐derived polyacetylene (PLA). The splitting fork over the deuterium peaks of CDCl_3_ indicates the quadrupolar doublet, whereas the central resonance (*) indicates the presence of an isotropic phase. The spectrum was recorded at 295 K (a). 1D ^13^C‐{^1^H}NMR spectrum of *R*‐indanol, which was measured in an 800‐MHz spectrometer equipped with a cryoprobe (b). For visual clarity of the isotropic and anisotropic signals of each carbon resonance, expansion of a part of the complete 1D carbon spectrum (c) in the range of 120–130 ppm is shown in (b). For each carbon, peaks with lower and higher intensities correspond to isotropic and anisotropic signals, respectively. The isotropic and anisotropic signals of carbons C5, C6, C4, and C7 are also labelled from left to right in (b). The photographic image of a sample in the NMR tube embedded in the biphasic PLA liquid crystal (d). The light‐yellow phase on the top corresponds to the isotropic phase, whereas the dark‐brownish phase in the lower half of the 3‐mm tube corresponds to the anisotropic phase [52]. (adapted from Ref. 52)

Recently, our group introduced the use of helically chiral PLA LLC media suitable for the enantiodifferentiation of chiral molecules. This PLA LLC media, which also exhibits a biphasic nature, offers several advantages over PBLG in a number of ways (Figure 3, right). We observed in a 17% (W_PLA_) sample in CDCl_3_ that PLA forms a biphasic mixture with both isotropic and anisotropic environments. This dual nature is evidenced by the ^2^H NMR spectra, showing a singlet from the isotropic phase alongside a quadrupolar doublet from the anisotropic phase and by the phase separation observed in the 3‐mm NMR tube (Figure 3, left and right, respectively).

Very recently, He et al. developed a new DMSO‐*d*_6_ compatible LLC system through the self‐assembly of C_21_H_43_–CONH‐V_4_K_3_–CONH_2_ [55]. They observed that the oligopeptide could rapidly form an LLC phase in DMSO‐*d*_6_, and after reaching a static equilibrium, this LLC phase underwent a slow settling process, ultimately leading to the formation of a biphasic LLC.

## 4 Experimental NMR Methods for RCSA Measurement

The pulse sequence used for the first report of ^13^C‐RCSA in small molecules was proton‐decoupled ^13^C NMR, which has a constant decoupling and a flip angle of 30° (*zgdc30*) [40], used for the epimeric discrimination of estrone. Later, Nath et al. used a variant of the same experiment but using a flip angle of 90° (*zgdc*) [41] to analyze several compounds using conventional stretching and compression devices. More recently, the use of sequences with power‐gated decoupling (*zgpg30*) was implemented [53]. Experiment parametrization in the implementation of ^13^C‐RCSA includes relaxation delays around 1.0–1.5 s, acquisition times between 0.9 and 1.5 s, and broadband Waltz‐16 ^1^H decoupling [56]. Despite the low natural abundance of ^13^C, measurement of ^13^C RCSAs is not restricted to high field spectrometers equipped with cryoprobes, if the signal‐to‐noise is maintained favorably. Navarro‐Vázquez and coworkers recorded ^13^C‐RCSAs on a spectrometer running at 400 MHz [57]. Measurement of ^1^H RCSAs is much more sensitive because of the high natural abundance and favorable gyromagnetic ratio of ^1^H and does not require complicated pulse sequences. The accurate measurement of carbon chemical shifts is essential for ^13^C‐RCSA, and different pulse sequences are employed to achieve this goal. The ^13^C‐{^1^H} NMR experiment using Ernst angle [102]—usually a 30° excitation angle—gives rise to the most intense signal in faster acquisition while doing signal averaging and is particularly beneficial for carbon detection for quaternary carbon–rich molecules. For most small molecules, a standard ^13^C‐{^1^H} NMR experiment with a 90° flip angle is sufficient. Alternatively, a power‐gated decoupled ^13^C NMR experiment with Ernst angle benefits from enhanced signal intensity through the NOE because of proton irradiation applied during the relaxation delay, making it especially useful for natural product chemists. Notably, there are no published reports of RCSA measurements using HSQC‐based experiments in small molecules, despite their successful applications in macromolecular systems.

In 2020, we introduced a rigorous approach to ^1^H RCSA measurement for the structural elucidation of minute amounts of small molecules [58]. We analyzed a series of natural compounds and showed how, by an improvement of the state of the art of RCSA methodologies, that is, by introduction of PMMA‐*d*_8_ [103] and an MSD (2.2/1.8 mm), it was possible to discriminate a few micrograms of strychnine (10 μg), (‐)‐α‐santonin (40 μg), and brucine (45 μg). The PMMA‐*d*_8_ reduced proton signals from the polymer significantly. The marginal polymer background of the nondeuterated material used in the preparation of PMMA‐*d*_8_ was suppressed by the spin echo NMR experiment without J modulation (PROJECT) [104]. It was also shown that liquid crystal made of PPA‐_L/D_‐Val_dec_ [105, 106] gives ^1^H resonances, which yield RCSA values usable for structural discrimination. Finally, by a combined analysis of ^1^H‐RCSA/ECD‐DFT, the configuration of two distant stereoclusters in briarane B3 could be determined on 35 μg of this marine natural product (vide infra). The reported configuration was confirmed later by X‐ray analysis [107]. Recently, the anisotropic potential of ^19^F was explored on several pharmacologically relevant chiral molecules dissolved in PBLG/CHCl_3_ [108]. Although a complete protocol for the use of ^19^F‐RCSAs in configurational assignment was not provided in that report, this first attempt highlights the potential of ^19^F‐RCSAs, particularly in terms of their sensitivity, anisotropy, and wide chemical shift dispersion. Variations in chemical shift were observed in ^19^F‐{^1^H} experiments on Flurbiprofen and Efavirenz. The authors emphasized the short experimental time (≈10 s) and the low detection limit (0.17 μmol mL^−1^). These results suggest that ^19^F‐RCSA‐based approaches could be especially valuable in cases where only very limited sample amounts are available. Until now, extracting ^15^N‐RCSAs for small molecules has remained a challenge for researchers, primarily because of their very low natural abundance. However, B. Luy demonstrated a proof of concept for ^15^N RCSA extractions using a stretchable γ‐PEO gel [83]. Although tested on ^15^N‐labeled ubiquitin, the experimental setup is applicable to small molecules, and the γ‐PEO gel has shown compatibility with structurally diverse natural products [91, 109]. At 60.774 MHz, 1D traces of HSQC spectrum reveal ^15^N‐RCSA values ranging from −13.8 to 14.7 Hz, indicating that incorporating ^15^N‐RCSA in the chemist's analytical toolkit is feasible.

Sample and magnetic field inhomogeneity are paramount to ensuring a uniform degree of analyte alignment along the NMR anisotropic sample. This translates into sharp observed resonances and a precise extraction of RCSAs. Alignment homogeneity in RCSA experiments can be easily visualized by observing the distribution of the ^2^H‐RQC, along the sample tube and its evolution as a function of swelling time, using a pseudo 2D pulsed field gradient–based deuterium NMR imaging experiment [76, 77]. This information would be lost if a conventional 1D ^2^H spectrum was used instead. The proposed experiments were applied to various alignment media, such as PBLG, PMMA, and PAN, allowing an unbiased evaluation of the quality of CCG and LLC samples for NMR measurements and detection of fractured CCGs. Using this approach, Chickmagalur et al. [45] showed the formation of the anisotropic phase over several days and confirmed the presence of minimal alignment inhomogeneity at the bottom of a sample of GO‐d‐CP liquid crystal [45]. The authors demonstrated that this did not pose a problem for the implementation of GO‐d‐CP liquid as a medium for NMR anisotropy.

### 4.1 RCSA A Priori Prediction

In 2022, Liu et al. achieved the a priori prediction of RCSAs through a mathematical framework designed for a parametrization protocol that deconvolutes an arbitrary alignment medium surface into several simple local landscapes [110, 111]. The authors determined the order parameters via a fitting procedure that maximizes the congruence between experimentally observed NMR parameters and their back‐calculated counterparts. The proposed method achieved RCSA prediction with a reasonable level of accuracy, while avoiding the need for a priori knowledge of the entire medium's morphology. This opens new avenues for understanding and utilizing RCSAs.

## 5 Chemical Shift Referencing

Referencing in RCSA extraction is performed to ensure that chemical shift differences reflect residual anisotropic effects rather than variations originating from sample or experimental conditions, such as small temperature changes or analyte concentration variations. Referencing the spectra to the nucleus with the lowest CSA in the compound studied seems to yield consistent results [40]. Yet, any nucleus can be used to reference, and indeed, Das et al. have shown how the discrimination capabilities of RCSAs on small molecules are independent of the selected reference nucleus, just changing the magnitude of the referenced RCSA [46]. It is advisable to use an external reference such as CCl_4_ [92], CDCl_3_ [92], or DMSO‐*d*_6_ [57] when the number of observable RCSAs is small. Some authors discourage the use of TMS [92], which, even having tetrahedral symmetry, still shows some anisotropy in PMMA/CDCl_3_ due to vibrational motion that destroys the tetrahedral symmetry.

### 5.1 Structural Elucidation of Small Molecules by RCSAs

**SCHEME 1:**
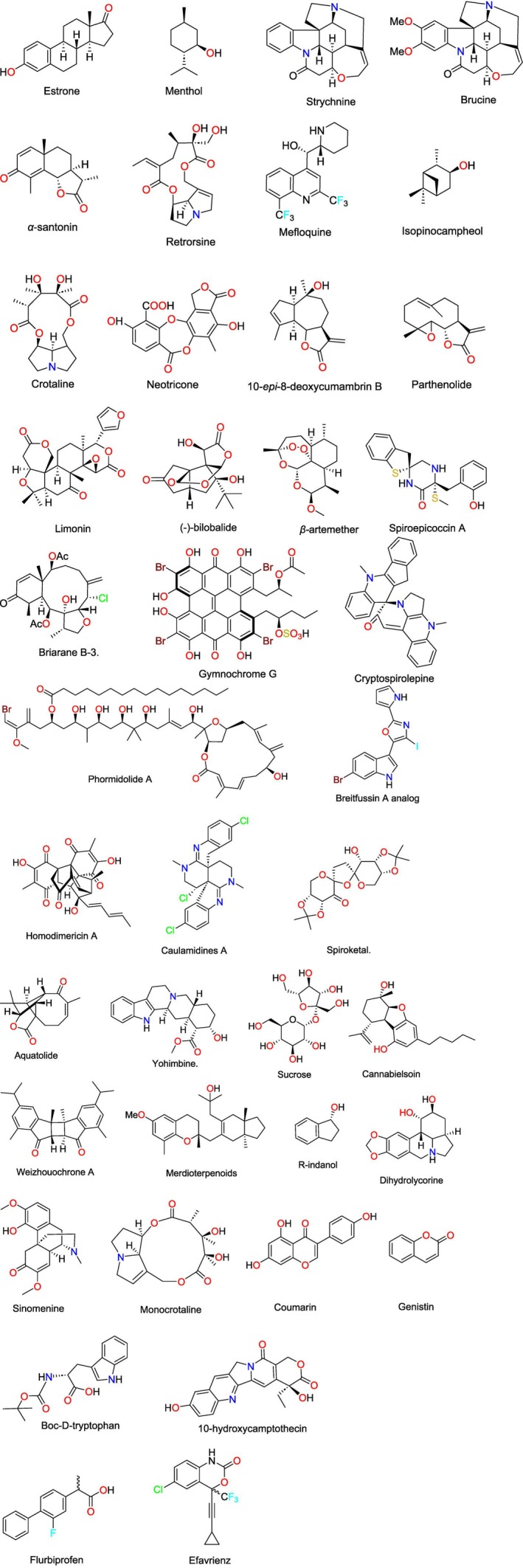
Molecular structures of synthetic and natural compounds that were structurally analyzed using RCSAs.

The study of small molecules based on RCSAs usually includes the determination of the relative configuration with the possibility of epimeric differentiation and diastereomeric differentiation, correction of resonance misassignment, conformational weight, structural revision, and absolute configuration when it is assisted by chiroptical/computational methods. The list of natural compounds studied using RCSAs is provided in Scheme 1 and Table 1.

**TABLE 1:** RCSAs in the structure elucidations of natural compounds.

Natural products	Source	Alignment medium/solvent	Year of study	Nuclei‐RCSA	Alignment method	Structural problem addressed	References
Strychnine	*Strychnos nux‐vomica* [112]	PDMS/CDCl_3_ [85] PPMA/CDCl_3_ [37, 113] PPMA‐*d*_8_/CDCl_3_ [58]; AAKLVFF/MeOH‐*d*_4_ [70]	2011/2016/2018/2019/2020	^13^C‐RCSA/^1^H‐RCSA (Proof of concept) [114]/^1^H‐RCSA (method development) [58]	VA‐NMR; SD; CD; MSD; Mod‐SD	Carbon misassignment/configuration assignment	[39, 41, 46, 58, 92]
Estrone	Endogenous hormones [115]	APS/DMSO‐*d*_6_ [116] PMMA/CDCl_3_ Poly‐HEMA	2011/2016/2019/2020	^13^C‐RCSA/^1^H‐RCSA	Kuchel; CD; SD; MCD; NMR tube	Differentiate two diastereomers	[32, 40, 41, 46, 53, 58]
Ubiquitin	Regulatory protein	γ‐PEO/D_2_O/H_2_O	2013	^15^N‐RCSA	Kuchel	Proof of concept	[83]
Mefloquine	Synthetic [6, 117]	PMMA/CDCl_3_	2016	^13^C‐RCSA	SD	Differentiate two diastereomers	[41]
Retrorsine	*Senecio jacobaea* [118, 119]	PMMA/CDCl_3_	2016/2020	^13^C/^1^H‐RCSA	SD	Configuration assignment of flexible molecule	[41, 58]
Menthol	Essential oils of Mentha species [120]	PMMA/CDCl_3_	2016	^13^C‐RCSA	SD	Configuration assignment	[41]
Cryptospirolepine	*Cryptolepis sanguinolenta* [121, 122]	Poly‐HEMA/DMSO‐*d*_6_ [123]	2017	^13^C‐RCSA/^1^ *D*_CH_	SD	Structural revision	[56]
Spiroketal	Rearrangement of a spiroketal molecule [124]	Poly‐HEMA/DMSO [123]	2017	^13^C‐RCSA	SD	Structural revision	[56]
Aquatolide	*Asteriscus aquaticus* [125, 126, 127]	Poly‐HEMA/DMSO‐*d*_6_ [123]	2017	^13^C‐RCSA	SD	Structural confirmation	[56]
10‐epi‐8‐deoxy‐cumambrin B	*Ambrosia confertiflora* [128]	PMMA/CDCl_3_	2018	^13^C‐RCSA	Mod‐SD	Configuration assignment	[48, 92]
Yohimbine	*Pausinystalia johimbe* [129]	PMMA/CDCl_3_	2018	^13^C‐RCSA	Mod‐SD	Configuration assignment	[92]
Caulamidines A and B	*Caulibugula intermis* [130]	Poly‐HEMA/DMSO‐*d*_6_	2018	^13^C‐RCSA	SD	Structural revision and AC	[130]
α‐Santonin	*Artemisia maritima* var. *stechmanniana* [131]	PMMA/CDCl_3_ PAN/DMSO PMMA‐*d*_8_/CDCl_3_ (40 μg)	2018/2019/2020	^13^C‐RCSA/^1^H‐RCSA	SD, MSD, CD	Configuration assignment	[48, 57, 58]
Brucine	*S. nux‐vomica* [132]	PMMA‐*d*_8_/CDCl_3_ (45 μg) PAN/DMSO‐*d*_6_O	2019/2020	^13^C/^1^H‐RCSA	SD, CD, MSD	Configuration assignment	[57, 58]
Briarane B3	*Briareum asbestinum* [107, 133]	PMMA‐*d*_8_/CDCl_3_ (35 μg)	2020	^1^H‐RCSA	MSD	Absolute configuration DFT‐ECD	[58]
Meroditerpene 1b	*Sargassum muticum* [134, 135]	PMMA‐*d*_8_/CDM	2022	^13^C‐RCSA	SMDC	Structural revision and AC (ECD/DFT)	[32]
(‐) Bilobalide	Ginkgo biloha L. [136, 137]	AAKLVFF/MeOH‐*d*_4_	2019	^13^C‐RCSA	Liquid crystal	Relative configuration assignment	[53]
Limonin	*Evodia glauca* Miq [138].	AAKLVFF/MeOH‐*d*_4_	2019	^13^C‐RCSA	Liquid crystal	Relative configuration assignment	[53]
β‐Artemether	*Artemisia annua L* [139]	AAKLVFF/MeOH‐*d*_4_	2019	^13^C‐RCSA	Liquid crystal	Relative configuration assignment	[53]
Spiroepicoccin A	*Epicoccum nigrum* [53]	AAKLVFF/MeOH‐*d*_4_	2019	^13^C‐RCSA	Liquid crystal	Relative configuration assignment	[53]
Parthenolide	*Chrysanthemum parthenium or Tanacetum parthenium* [140]	PBLG/CDCl_3_/DMSO‐*d*_6_	2019	^13^C‐RCSA	Liquid crystal	Relative configuration assignment	[51]
Sucrose		PBLG/CDCl_3_/DMSO‐*d*_6_	2019	^13^C‐RCSA	Liquid crystal	Relative configuration assignment	[51]
Neotricone	Lichen genus *Phaeographis* [141]	PBLG/CDCl_3_	2020	^13^C‐RCSA	Liquid crystal	^13^C‐NMR assignment revision	[99]
Gymnochrome G	*Hypalocrinus naresianus* [96]	Magnetically induced self‐alignment/CD_3_OH	2020	^13^C‐RCSA	Magnetic alignment	Relative configuration assignment	[96]
Phormidolide	Cyanobacterium *Leptolyngbya* sp. [142]	PBLG/CDCl_3_	2020	^13^C‐RCSA	Liquid crystal	Relative configuration assignment	[143]
Breitfussin A	*Thuiaria breitfussi* [9]	Poly‐HEMA/MMA/DMSO‐*d*_6_	2020	^13^C‐RCSA	Liquid crystal	Constitutional assignment	[144]
Cannabielsoin	*Cannabis sativa* [145]	PBLG/CDCl_3_	2021	^13^C‐RCSA	Liquid crystal	Structural revision	[145]
Weizhouochrone A	*Anthogorgia ochracea* [71]	AAKLVFF/CD_3_OD	2022	^13^C‐RCSA	Liquid crystal	Relative configuration assignment	[71]
R‐indanol	Synthetic	L‐valine‐derived helically chiral polyacetylenes/CDCl_3_	2023	^13^C‐RCSA	Liquid crystal	Enantiomeric differentiation	[52]
(+)‐IPC	Synthetic	L‐valine‐derived helically chiral polyacetylenes/CDCl_3_	2023	^13^C‐RCSA	Liquid crystal	Enantiomeric differentiation	[52]
Dihydrolycorine	*Lycoris radiata* [146]	(FK)_4_ oligopeptides/D_2_O	2024	^13^C‐RCSA	Liquid crystal	Relative configuration assignment	[54]
Sinomenine	*Sinomenium acutum* [147]	(FK)_4_ oligopeptides/D_2_O	2024	^13^C‐RCSA	Liquid crystal	Relative configuration assignment	[54]
Monocrotaline	*Crotalaria spectrabilis* [148]	(FK)_4_ oligopeptides/D_2_O	2024	^13^C‐RCSA	Liquid crystal	Relative configuration assignment	[54]
Coumarin		Biphasic C_21_H_43_–CONH‐V_4_K_3_–CONH_2_ LLC/DMSO‐*d*_6_	2024	^13^C‐RCSA	Liquid crystal	Constitution	[54]
Genistein	*Glycine max* [149]	Biphasic C_21_H_43_–CONH‐V_4_K_3_–CONH_2_ LLC/DMSO‐*d*_6_	2024	^13^C‐RCSA	Liquid crystal	Constitution	[54]
Boc‐D‐tryptophan	Synthetic	Biphasic C_21_H_43_–CONH‐V_4_K_3_–CONH_2_ LLC/DMSO‐*d*_6_	2024	^13^C‐RCSA	Liquid crystal	Constitution	[54]
10‐hydroxycamptothecin	*Camptotheca acuminata* [150]	Biphasic C_21_H_43_–CONH‐V_4_K_3_–CONH_2_ LLC/DMSO‐*d*_6_	2024	^13^C‐RCSA	Liquid crystal	Constitution	[54]
*R/S‐*Flurbiprofen	Synthetic	PBLG	2024	^19^F‐RCSA	Liquid crystal	Enantiomeric analysis	[108]
*R/S*‐Efavrienz	Synthetic	PBLG	2024	^19^F‐RCSA	Liquid crystal	Enantiomeric analysis	[108]

*Note:* NMR tube: liquid crystals create alignment directly inside the NMR tubes; magnetic alignment: created using several magnetic fields [96].

Abbreviations: CD: compression device [37]; Kuchel: rubber‐based stretching apparatus [91]; Mod‐SD: modified gel‐stretching device [92]; MSD: microstretching device [58]; SD: stretching device [81]; SMCD: semimicro‐compression device [32]; VA‐NMR: variable angle NMR [39].

For structure elucidation, the measured RCSAs are fitted by singular value decomposition (SVD) into chemical shift anisotropy tensors of the studied molecule to determine the five independent order parameter variables ( $A_{\mathrm{ij}}$) [151]. Then, RCSAs are back‐calculated and compared with their experimental counterparts. The agreement between the experimental and calculated RCSAs is expressed in terms of a *Q* factor [152] defined as follows:

(11) $$Q=\sqrt{\frac{\sum_{i}{\left(∆{\text{RCSA}}_{i}^{\exp}-∆{\text{RCSA}}_{i}^{\text{theo}}\right)}^{2}}{\sum_{i}{\left(∆{\text{RCSA}}_{i}^{\exp}\right)}^{2}}}.$$

Regardless of the accuracy, the SVD procedure ensures optimal consistency between the test data and experimental data, yielding *Q* factor for each candidate structure, considered for analysis. The correct structure should possess the best agreement between experimental and calculated RCSAs and thus exhibit the lowest *Q* factor compared to the incorrect candidates. In some cases, the *Q* factor fails to provide any structure distinction; then, the CSA‐size‐based *Q* (*Q*_CSA_) factor, as defined below, can be used:

(12) $$Q_{\mathrm{CSA}}=\sqrt{\frac{\sum_{i}{\left(\left(∆{\text{RCSA}}_{i,\mathrm{ax}}^{\exp}-∆R{\mathrm{CSA}}_{i}^{\text{theo}}\right)/{\mathrm{CSA}}_{i,\mathrm{ax}}\right)}^{2}}{\sum_{i}{\left(∆{\text{RCSA}}_{i}^{\exp}/{\mathrm{CSA}}_{i,\mathrm{ax}}\right)}^{2}}.}$$

The RDC‐based stereochemical analysis takes into account that one‐bond ^1^H−^13^C bond length is very stable, and therefore, the dipolar coupling is indifferent to the specific carbon or hydrogen involved. Different ^1^H–C bond vectors are equally weighted in the *Q* factor–based analysis [41]. In contrast, CSA tensors vary widely, particularly between sp^3^ and sp^2^ carbons and protons, and the sizes of CSAs of sp^2^ carbons are 4–10 times larger than those of sp^3^ carbons. Because the configuration of a molecule is encoded in the network of all carbons and protons, the ideal *Q* value should scale the CSA values to the same size so that only orientation information is reflected. Toward this goal, we employed the following procedure: First, the alignment tensor is derived by fitting all RCSAs to the DFT‐computed CSA tensor through the SVD method [151]. Then, a new quality factor, *Q*_CSA_, is calculated by scaling both experimental and back‐calculated RCSAs by corresponding atoms' chemical shift anisotropies, using the formula given in Equation (12), where CSA_*i*,*ax*_ equals *σ*_33_ − (*σ*_22_ + *σ*_11_)/2 and the chemical shielding eigenvalues *σ*_11_, *σ*_22_, and *σ*_33_ are obtained from DFT.

Strychnine, a neurotoxic monoterpene indole alkaloid, was first isolated from the seeds of *Strychnos nux‐vomica* [153]. It is a highly rigid molecule with at least one marginally populated second conformation observed by NOE contacts and confirmed by RDC studies [154, 155, 156, 157]. Several RCSA studies have been conducted: In 2016, Nath et al. [41] achieved configuration assignment using both a stretching and compression device (Figure 1a, and 1f). Using a sample mass of 14 mg in a New Era's (https://newera‐spectro.com/%20%20) 4.2/3.2‐mm tube, they found the ^13^C‐RCSA range between −1.8 and 10.5 Hz, in a spectrometer running at 600 MHz, whereas the compression device sample was of 7.5 mg, and the uncorrected ^13^C‐RCSA spread around −29.3 and 2.9 Hz, observed at 700 MHz. Both experiments used a PMMA matrix to impose the necessary degree of alignment onto the analyte. In both cases, the lowest Cornilescu's *Q* factor selected the correct configuration (*RSSRRS*) among the 13 energetically feasible configurations. Nath et al. included a statistical error analysis in the assignment of strychnine by ^13^C‐RCSA.

**FIGURE 4:**
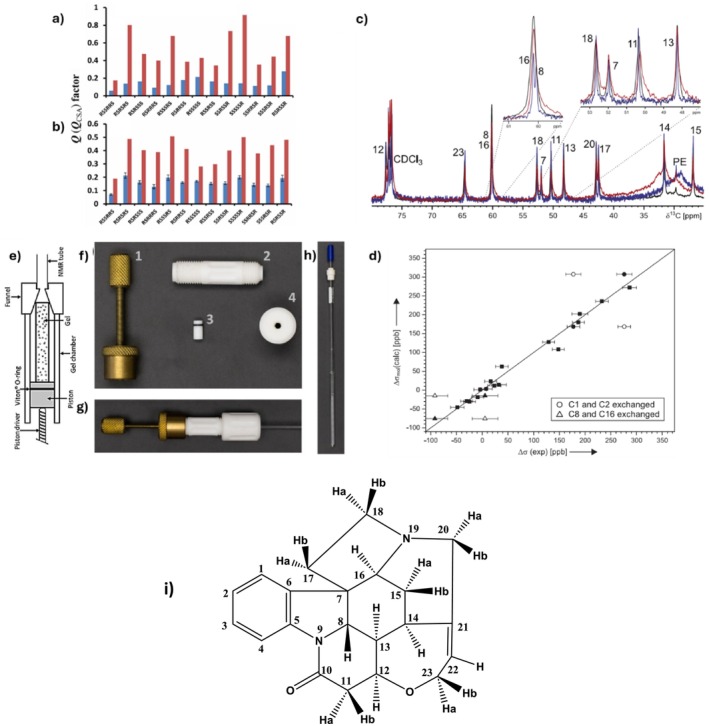
The quality of the fit is expressed as a quality factor (*Q*) where the correct structure is expected to have the lowest *Q* value. (a) Results from the stretching device: *Q* (blue) and *Q*_CSA_ (red) factors calculated for the lowest energy structures of 13 possible configurations from DFT calculation using only ΔRCSAs. (b) Results from the compression device. Statistical error is calculated by removing 10% of the data randomly and calculating the *Q* values 10 times [41]. (c) Aliphatic region of ^13^C‐proton decoupled 1D spectra measured on strychnine diffused into a stretched PDMS/CDCl_3_ gel at *θ* = 54.7° (black), *θ* = 65° (red, light gray), recorded in a 4‐mm MAS probehead, and *θ* = 0° (blue and dark gray), recorded in a 500‐MHz TXI triple resonance liquid‐state probe was employed to acquire high‐resolution spectra in a conventional 5‐mm NMR tube. NMR signals originating from the poly (ethylene) plug are marked with PE. Spectra are referenced to C13/C14/C15 as the carbons with the smallest RCSA tensors [39] (d). Backcalculated Δ*σ*_mol_ (calc) [ppb] versus experimental Δ*σ*_exp_ [ppb] residual chemical shift anisotropies for strychnine in PDMS/CDCl_3_. The accidental misassignment of C1 and C2 (open and filled circles) could be identified by the plot shown. In the same way, carbons C8 and C16, which overlap in the isotropic spectrum, can unambiguously be assigned (open and filled triangles). RCSA referencing was achieved by the average uncorrected RCSA value for C13, C14, and C15 as the three nuclei with the smallest theoretically determined CSA tensor [39]. (e) Modified stretching device [92]. (f) Representation of the unassembled device: 1. piston driver, 2. gel chamber, 3. piston with Viton O‐ring, and 4. funnel. (g) Assembled device. (h) Stretched gel in a 4‐mm tube inside the conventional 5‐mm tube. A Teflon plug made from Teflon tape is inserted into the bottom open end of the 4‐mm tube to prevent the presence of isotropic solution between the inner wall of the 5‐mm tube and the outer wall of the 4‐mm tube [92]. (i) Molecular structure of strychnine with atomic numbering. (adapted from refs. 39, 41, 92.)

Grit Kummerlöwe and Burkhard Luy studied strychnine using both a magic angle sample spinning (MAS) probehead of 4 mm and a variable angle sample spinning (VASS) at different tilt angles (*θ*). By varying the alignment angle of the stretching axis of the mechanically stretched polymer gel relative to the static magnetic field, VASS minimizes inconsistencies often caused by isotropic variations or shifts in temperature across phase transitions (Figure 4c); resulting spectra had a linewidth of around 8 Hz, which is in part due to the VASS probes not being optimized for liquid state measurements. For precise chemical shift calibration, the authors examined several referencing methods, including one based on tetramethylsilane (TMS) [39]. Despite theoretical assumptions, they found the TMS correction insufficient, resulting in suboptimal correlation between experimental and calculated RCSA values. Thus, they used an empirical approach, focusing on carbons with minimal CSA (C13, C14, and C15; see Figure 4h). If chemical shifts of carbons with small CSA are accessible, their uncorrected average RCSA could approximate the reference shift upon alignment. Strychnine was also employed as a chemical model for the refinement of a modified version of the stretching device (Mod‐SD) by Erich Hellemann and Roberto R. Gil [92]. Components of the Mod‐SD (Figure 3e–h) facilitate stable stretching without the need for separate sections within the tube, which minimizes sample manipulation and optimizes consistency in alignment strength. The strychnine analysis yielded a *Q* factor of 0.082 against 0.116 for the *RSSSRS* stereoisomer. In comparison, using the conventional stretching device, Nath et al. [41] observed that the correct configuration has a considerably lower *Q* of 0.050 to differentiate it from the closest incorrect configuration of *Q* = 0.100 corresponding to the *RSRRRS* configuration [41].

The ^13^C‐RCSAs methodology was also employed to elucidate the relative configuration of the heterocyclic marine alkaloid caulamidine A [130]. Its structure is a combination of piperidine rings with substituted aromatic rings (with chlorine) and chiral centers in a complex arrangement. Initially, its constitution and relative configuration were proposed by a combination of LR‐HSQMBC [143, 158], 1,1‐HD‐ADEQUATE [122, 159, 160], and NOE experiments and a set of conventional 1D/2D NMR experiments. Part of the challenge in caulamidine A study was the identification of chlorine position. It was achieved by observing the ^35/37^Cl isotope effect for C‐7 (Ring C), C‐11 (Ring D), and C‐19 (Ring F), thanks to the implementation of the sensitive bs‐CLIP‐HSQMBC [161].

**FIGURE 5:**
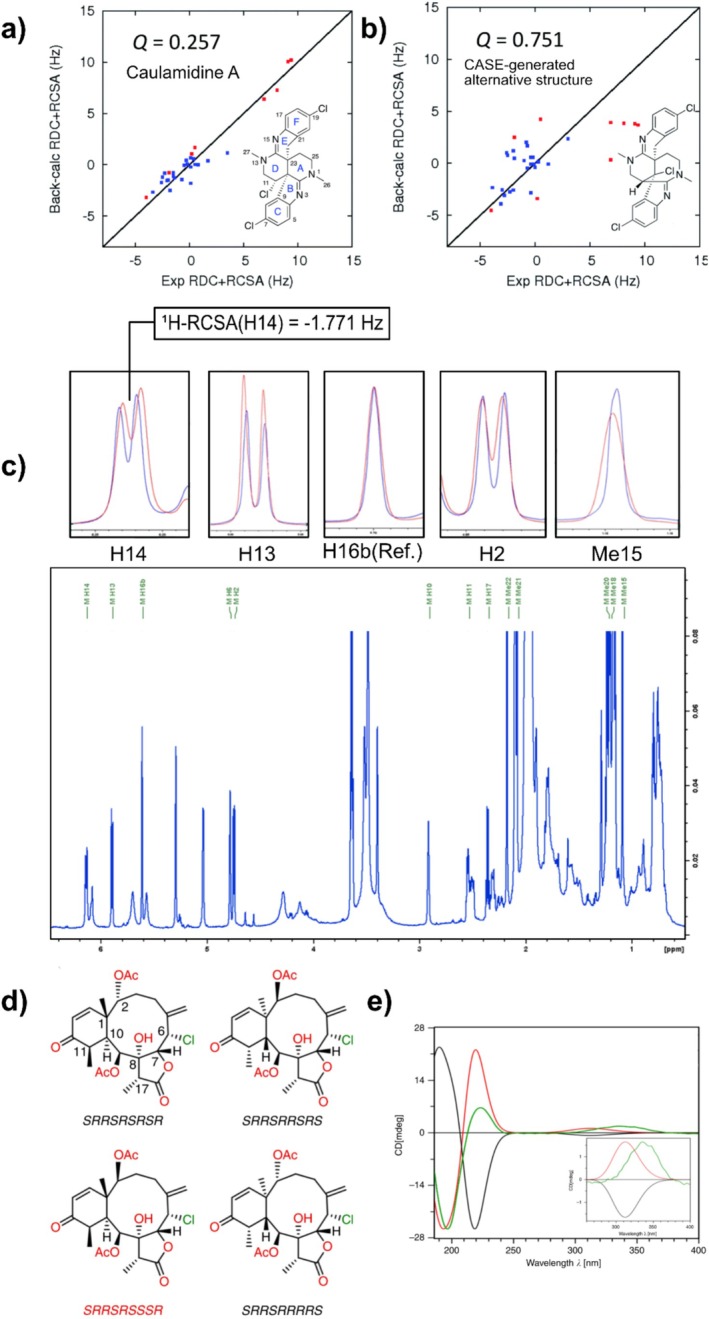
Comparison of the experimental versus DFT‐calculated RDCs (red) and RCSAs (blue) values for caulamidine A (a) and the CASE‐generated alternative structure (b) [130]. 1D ^1^H spectrum for a 35‐μg sample of briarane B‐3 in PMMA‐*d*_8_ (70/0.05) analyzed in a microstretching device (2.2/1.8 mm). (c) ^1^H RCSAs for five chosen protons: H14, H13, H16b (Ref.), H2, and Me15. Spectra in blue and red color codes were obtained in isotropic and anisotropic conditions, respectively (top race). Analysis was done in a Bruker spectrometer running at 800 MHz. (*zg*; number of scans: 1024) (d). The four possible diastereomers for the structure of briarane B3, of which the *SRRSRSSSR* (red) is found to be the correct one (e). Calculated ECD spectra of *SRRSRSSSR* (red line) and enantiomer *RSSRSRRRS* (black line) versus experimental ECD spectrum (green line) of briarane B3. Note that the ordering of the stereocenters is C6, C7, C8, C9, C17, C1, C2, C10, and C11 (e) [58]. (adapted from refs. 57 and 130.)

An attempt to confirm the results by CASE analysis, using a restricted set of NMR input data, provided an ambiguous result, with two equally probable structures (see Figures 5a and 5b) [130]. The ultimate verification of caulamidine A's constitution was achieved by a combination of ^13^C‐RCSA and ^1^ *D*_CH_ analysis. The molecule was aligned in poly‐HEMA gel in a stretching device (4.2/3.2 mm); 21 up to 24 RCSAs were measured with the ^13^C‐proton decoupled 1D experiment with a recycling delay of 1.5 s, and an acquisition time of 0.55 s in a spectrometer equipped with a Prodigy probe. This analysis showed the lowest *Q* and higher fitting linearity for *SSS*‐caulamidine A‐1 (Figure 5a), conclusively confirming the structure assigned to caulamidine A.

Molecules from natural sources are usually available in scarce amounts. Polymeric alignment media introduce line broadening and background residual signals. Consequently, molecules in the range of a few micrograms were not possible to be studied. To overcome this situation, Griesinger et al. have developed the ^1^H‐RCSA methodology [58], which, by combining deuterated PMMA and microstretching devices, allowed us to significantly reduce the sample amount required. The deuterated PMMA was polymerized in a 1.5‐mm capillary tube, and after washing with deuterated solvent, it was inserted into the 2.2/1.8‐mm microstretching device, manufactured by Hilgenberg (https://www.hilgenberg‐gmbh.de/), in only a few microliters of CDCl_3_. Sharp lines and acceptable signals from the gel due to a high level of deuteration were observed in the 1D ^1^H. The remaining background signal, coming from the radical initiator and cross‐linker that were not deuterated, was removed by employing Spin echo NMR spectra without J modulation experiments (Figure 4c) [104]. Combined ^1^H‐RCSA/DFT‐ECD was successfully utilized in the determination of the absolute configuration of 35 μg of briarane B3. Briarane B3 was isolated from the gorgonian *Briareum asbestinum* [133]. It contained nine stereocenters, C1, C2, C6, C7, C8, C9, C10, C11, and C17, where two stereoclusters were first defined by NOE/J‐based NMR analysis, using only 35 μg for the isotropic spectra in a 1.7‐mm NMR capillary tube. Employed 2D experiments were speeded up with NUS [162]. The analysis allows the determination of the configuration at C6, C7, C8, C9, and C17 as *SRRSR* or *RSSRS*. Further NMR analysis reduced the possible configurations at C1, C2, C10, and C11 to four, that is, *SRSR*, *SSSR*, *RRRS*, and *RSRS*. Thus, isotropic NMR parameters simplify the problem: The 256 possible configurations of briarane B3 can be reduced to 4, which were *SRRSRSRSR*, *SRRSRRSRS*, *SRRSRSSSR*, and *SRRSRRRRS*, for C1, C2, C6, C7, C8, C9, C10, C11, and C17, respectively, and their enantiomers. Different diastereoisomers were independently evaluated by ^1^H‐RCSAs; 35 μg of briarane B3 were oriented in PMMA‐*d*_8_, and experimental RCSAs ranged from 1.8 to 3.2 Hz at a ^1^H frequency of 800 MHz. Proton CSAs were computed using DFT at mpw1pw91/6‐311+G(2d,p). Data were fitted using a pool of conformers using a single tensor. This was done for the four relative configurations, of which the *SRRSRSSSR* briarane B3 configuration furnished the lowest *Q* (*Q*_CSA_) factor of 0.176 (0.219) ± 0.036 (0.047) (Figure 4d). The comparison of the experimental CD spectrum of briarane B3 and DFT‐computed ECD spectra of both enantiomers of the relative configuration *SRRSRSSSR* yielded the absolute configuration of briarane B3. The ECD spectra were computed using conformer populations weighted by *Q* minimization through ^1^H‐RCSA analysis (Figure 5e). During the extraction of ^1^H‐RCSAs using PPA LC, line broadening is indeed generally observed, and in some cases, certain resonances may be completely occluded by the liquid crystal background signal. Nevertheless, the degree of alignment imposed by PPA LCs was sufficient to generate measuable ^1^H‐RCSAs. We note that the observed line broadening does contribute to noise in the extracted RCSAs. Protonated CCGs represent a better option than LCs for measuring ^1^H‐RCSAs, because they exhibit much lower line broadening than the studied LCs. Furthermore, the use of fully deuterated PMMA eliminates the polymer background and provides spectra with resonances that show almost no distortion. Certainly, the extent of proton distortion depends on proton density; however, a reasonable proportion of the resonances remain unaffected by broadening. An example of the extraction of ^1^H‐RCSAs is shown in Figure 5c [58], and the experimental setup is illustrated in the accompanying video on our YouTube channel (https://www.youtube.com/watch?v=z2THX9oVVwg). Additionally, pure shift experiments (e.g., PSYCHE) can indeed be considered for recording RCSAs in highly coupled spin systems, provided that the sample concentration is sufficiently high. However, PSYCHE experiments typically take advantage of ~20% of the signal relative to conventional experiments. Thus, recording a ^1^H pure shift experiment on a ~30‐μg sample would be impractical in most laboratories, even when using a 700‐MHz spectrometer. In summary, while ^1^H‐RCSAs are affected by the issues as mentioned, they remain highly sensitive anisotropic NMR observables. Importantly, they can be used independently to address structural problems. At present, their combined use with other observables, such as ^13^C‐RCSAs, remains an open opportunity for the community to explore further.

**FIGURE 6:**
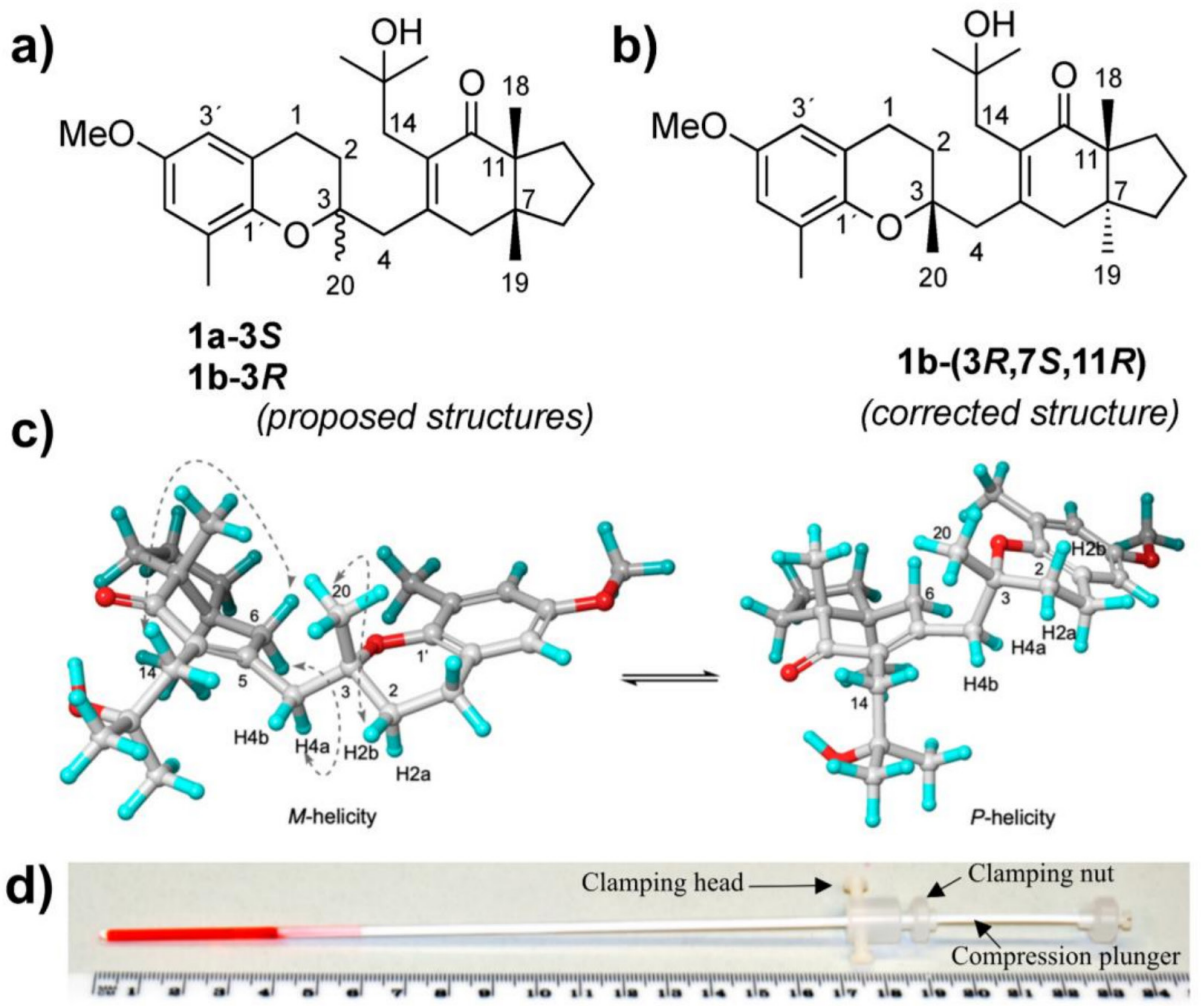
Structures of the meroditerpenoids of the meroditerpenoids 1a and 1b (a and b). *P*‐ and *M*‐helicities equilibrium present on meroditerpene‐1b, observed by NMR isotropic techniques (NOE/J‐BCA) and confirmed by RCSA study (c). Semimicro‐compression device prototype used during the recording of NMR anisotropic parameters of meroditerpene‐1b (d). Complete alignment system shown here includes a common 3‐mm NMR tube, compressed PMMA (75/0.25) gel stick swollen in CDCl_3_, and the semimicro‐compression apparatus (d). Compression device consists of a clamping head/nut and fastening screws, which keep the plunger attached to the external walls of the 3‐mm NMR tube when the gel stick is under compression. Gel shown was colored using a pigment from Faber‐Castell. (adapted from ref. 32.)

Finally, we would like to draw the readers' attention to epimer differentiation using RCSA. Epimer differentiation in natural product chemistry is a problem recurrently addressed by chemists. Discrimination between estrone and *epi*‐estrone has been successfully conducted by RCSAs because of their early applications to structural elucidation problems [40]. The structure of the meroditerpene‐1b, isolated from *Sargassum muticum* [134, 135], was recently revised using RCSAs [32]. It is a tetraprenyltoluquinol chromane, which exhibits a moderate degree of flexibility, featuring two stereoclusters separated by four covalent bonds. The configuration of the stereocenters at Position 3, in the chromane moiety, was initially misassigned together with the configuration of C7, in the hydrindane skeleton (Figure 6a). Connection among the chiral centers was achieved by combining isotropic and RCSA methodologies (Figure 6b) [32]. The main challenges faced were disentangling the helicity equilibrium observed in the chromane fragment and its contribution to the ECD spectrum, as well as the molecular rotation around C4. These challenges were addressed by ^13^C‐RCSA (Figure 6c) [163, 164]. ^13^C‐RCSAs were observed in a semimicro‐compression device prototype (PMMA‐*d*_8_/DCM) (Figure 6d). The analysis assigned the relative configuration of meroditerpene‐1b based on the small *Q* (QCSA) = 0.079(0.299) for 3R*7S*11R*‐1b compared to 0.156(0.341) for 3R*7S*11*R*‐1b. DP4‐based methods confirm in this case the assignment of the relative configuration of meroditerpene‐1b. The absolute configuration of meroditerpene‐1b was determined as 3R7S11R by combining two chiroptic methods, namely, ECD and OR.

Das et al. [46] conducted a systematic study on how chemical shift anisotropy tensors calculated with different DFT levels and basis sets impact the RCSA results. It is found that results remain invariant to the type of DFT functional and basis set used. A benchmark of DFT in forecasting carbon chemical shift anisotropy tensors for the interpretation of the RCSA data was later published by Ketzel et al. [165]. Semilocal DFT approaches are also found to be capable of analyzing RCSA data for conformation and configuration determination, which lowers computing costs in comparison to the widely used hybrid method, like B3LYP [165].

## 6 Conclusions

The use of RCSAs to determine the constitution and configuration of small molecules in orienting media has significantly advanced over the past decade and continues to be applied by an increasing number of research groups. Throughout this review, we introduced the theory of anisotropic NMR, discussed NMR experiments for anisotropic samples, walked through a typical workflow of RCSA extraction and analysis, and summarized recent advancements in alignment media for solving structural problems of complex natural products. As an evolving field, there is still a lot of space for further advancements. The widespread application of this technology is limited by the fact that not all alignment media are commercially available, although several important ones such as PBL/DG, PEL/DG, PCBL/DL (polyglutamates), and PLA (poly‐L‐Val‐dec) and polymeric gels such as PAN and PBLG are accessible. Therefore, efforts should be made to continue developing novel alignment media that commercial vendors would distribute. By leveraging approaches from synthetic organic chemistry and polymer science, new alignment media can be systematically designed, especially chiral variants with enantiodifferentiation capabilities and deuterated versions of alignment media for proton RCSA measurements. Further efforts can be made to synthesize liquid crystalline media with a biphasic nature.

The growing field of RCSAs will continue to make significant contributions to a wide range of structural determination challenges, becoming an integral tool for all NMR spectroscopists. Obviously, anisotropic NMR remains dynamic and highly relevant today. We hope that this review offers readers the necessary context and background information and potentially new ideas in this area.

## Funding

N.N. gratefully acknowledges the financial support by the Science and Engineering Research Board, New Delhi, for CRG Grant with File No: CRG/2022/005993. C.G. was funded by the Max Planck Society.
